# Chemical Acetylation of Ligands and Two-Step Digestion Protocol for Reducing Codigestion in Affinity Purification–Mass Spectrometry

**DOI:** 10.1021/acs.jproteome.3c00424

**Published:** 2023-09-15

**Authors:** David M. Hollenstein, Margarita Maurer-Granofszky, Wolfgang Reiter, Dorothea Anrather, Thomas Gossenreiter, Riccardo Babic, Natascha Hartl, Claudine Kraft, Markus Hartl

**Affiliations:** † Department for Biochemistry and Cell Biology, University of Vienna, Center for Molecular Biology, Vienna Biocenter Campus (VBC), Dr. Bohr-Gasse 9, Vienna 1030, Austria; ‡ Mass Spectrometry Facility, Max Perutz Laboratories, Vienna Biocenter Campus (VBC), Dr. Bohr-Gasse 7, Vienna 1030, Austria; § St. Anna Children’s Cancer Research Institute (CCRI), Zimmermannplatz 10, Vienna 1090, Austria; ∥ Institute of Biochemistry and Molecular Biology, ZBMZ, Faculty of Medicine, University of Freiburg, Freiburg 79104, Germany; ⊥ Faculty of Biology, University of Freiburg, Freiburg 79104, Germany; # Spemann Graduate School of Biology and Medicine (SGBM), University of Freiburg, Freiburg 79104, Germany; ∇ CIBSS - Centre for Integrative Biological Signalling Studies, University of Freiburg, Freiburg 79104, Germany

**Keywords:** mass spectrometry, proteomics, affinity purification, immunopurification, nanobody, GFP, streptavidin, Sulfo-NHS-Acetate, chemical acetylation, ligand

## Abstract

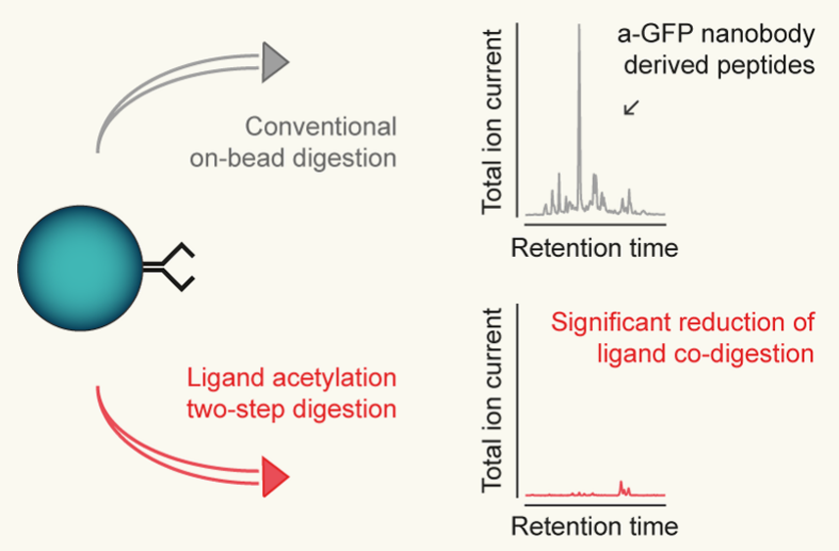

We present an effective, fast, and user-friendly method to reduce codigestion of bead-bound ligands, such as antibodies or streptavidin, in affinity purification-mass spectrometry experiments. A short preincubation of beads with Sulfo-NHS-Acetate leads to chemical acetylation of lysine residues, making ligands insusceptible to Lys-C-mediated proteolysis. In contrast to similar approaches, our procedure offers the advantage of exclusively using nontoxic chemicals and employing mild chemical reaction conditions. After binding of bait proteins to Sulfo-NHS-Acetate treated beads, we employ a two-step digestion protocol with the sequential use of Lys-C protease for on-bead digestion followed by in-solution digestion of the released proteins with trypsin. The implementation of this protocol results in a strong reduction of contaminating ligand peptides, which allows significantly higher amounts of sample to be subjected to LC–MS analysis, improving sensitivity and quantitative accuracy.

## Introduction

Affinity purification coupled to mass spectrometry (AP–MS) is a widely employed approach to investigate protein–protein interactions and analyze the composition of protein complexes. In AP–MS, a protein of interest, also called the bait, is isolated from a biological sample using affinity purification and is subsequently subjected to liquid chromatography–mass spectrometry (LC–MS) analysis. In standard AP–MS workflows, antibodies coupled to agarose or magnetic beads are often used for the purification of native or epitope tagged bait proteins, utilizing, for example, FLAG, HA (human influenza hemagglutinin), or GFP (green fluorescent protein) tags. Recently, nanobodies have been developed, which are advantageous for AP–MS due to their high stability and small size.^1^ For example, anti-GFP nanobody-coated beads, with a particularly high affinity for GFP-tags, have been demonstrated to be a valuable tool for investigating the interactomes of GFP-tagged proteins.^2^ Additionally, other types of protein–ligand interactions can be employed as an alternative to antibody-based affinity purification, such as the streptavidin–biotin interaction, which exhibits a remarkably high affinity and tolerates fully denaturing conditions. Streptavidin beads have therefore been used in a number of specialized AP–MS workflows, such as purification of bait proteins under denaturing conditions to preserve post-translational modifications,^3^ cross-linking MS,^4^ and in vivo proximity labeling techniques such as BioID and APEX.^5,6^

Regardless of the ligand used, AP workflows for bottom up MS typically consist of three key steps: (1) binding of the bait protein to an immobilized ligand, (2) washing of the affinity matrix to remove nonspecifically bound proteins, and (3) elution of bound bait proteins to enable downstream analysis of the sample via LC–MS. Proteins can be eluted by competition with a soluble agent or by disrupting ligand–bait interactions using acidification or strong detergents. Alternatively, proteins can be released by enzymatic digestion while still being bound to the matrix, which is referred to as on-bead digestion. In this approach, proteins bound to the affinity matrix are reduced, alkylated, and then incubated with a protease. As a result, enzymatically cleaved peptides are directly released from the affinity matrix into the solution. On-bead digestion is often the method of choice, as it integrates well into LC–MS workflows and has been reported to reduce sample losses and increase sensitivity.^7,8^

One of the challenges associated with on-bead digestion is the codigestion of ligand molecules. This can lead to ligand-derived peptides dominating the ion chromatogram, as the ligand typically constitutes the majority of proteins bound to the affinity matrix. Significant contamination of the sample with ligand-derived peptides compromises the detection of low abundant proteins either by suppressing coeluting peptides or by limiting the amount of sample material that can be injected into the LC–MS system.

This problem has been recognized by us and others, specifically when utilizing streptavidin or anti-GFP nanobody-coated beads for AP–MS.^9,10^ In our routine operations at the core facility, we have occasionally observed that the contamination with peptides derived from the ligand represents the most prominent signals in the LC–MS analysis. This has been especially observed in AP–MS experiments, in which the beads were only partially saturated with the bait. Another example relates to BioID experiments, because the relative amount of streptavidin can be high as compared to the biotinylation signal that is dispersed over numerous proteins.

To address this problem, two approaches have been proposed to create a protease-resistant ligand. The first involves genetic engineering of the ligand to remove the amino acids lysine and arginine that are targeted by tryptic proteolysis. This strategy necessitates significant effort and expertise and cannot be applied to commercially available products, which makes it impractical for general application. The second approach entails chemical derivatization of arginines and lysines to protect them from digestion. However, the available protocols are time-consuming and require the use of multiple toxic and nonstandard chemicals.^10,11^ Additionally, the ligand becomes exposed to a harsh chemical environment, potentially compromising its epitope binding capacity.

Here, we present an optimized on-bead digestion protocol that drastically reduces the codigestion of ligands while being simple, fast, and using nontoxic chemicals only. Pretreating the ligand-coated beads with Sulfo-NHS-Acetate (S-NHS-Ac) for 1 h at neutral pH and room temperature efficiently acetylates lysine residues, making the ligand proteolytically resistant to the Lys-C protease. Lys-C treatment then allows efficient elution of the bait protein with minimal contamination from ligand-derived peptides. After removal of the beads, the eluate can be further digested in a second step using trypsin. We demonstrate the successful application of this bead-acetylation two-step digestion protocol with anti-GFP nanobody and streptavidin-coated magnetic beads, two widely used systems for AP–MS.

## Experimental Procedures

### Yeast Strains and Growth Conditions

The following *S. cerevisiae* strains were used in this study: yCK566 (wild type; BY474x; Mat a; obtained from Euroscarf) and yTB281 (sfGFP-ATG8; BY474x; Mat A; described in ref (12)). Yeast cells were grown in rich medium (YPD, 1% yeast extract, 2% peptone, and 2% glucose) to mid log phase subsequently treated with 220 nM rapamycin for 30 min to induce autophagy. Yeast cultures were incubated with shaking (220 rpm) at 30 °C.

### Mammalian Cell Lines, Cell Culture Conditions, and Stable Cell Line Generation

The following mammalian cell lines were used in this study: cRB7 (2xFKBP-APEX2-ULK1; HEK293T Flp-In T-REx; Hygromycin and Blasticidin resistance; described in this study) and cRB12 (2xFKBP-APEX2; HEK293T Flp-In T-REx; Hygromycin and Blasticidin resistance; described in this study). cRB7 and cRB12 were created by integration of Flp-In expression vectors into HEK293T Flp-In T-REx (R78007, Thermo Fisher Scientific) cells.

HEK293T Flp-In T-REx cells were grown in incubators at 37 °C, supplied with 5% (v/v) CO_2_-air atmosphere in DMEM (Dulbecco’s modified Eagle’s medium; Sigma-Aldrich, D6429) containing 10% FBS (fetal bovine serum, Sigma, F7524-500 ML), 5 U/mL penicillin (Sigma, P4333-100 ML), and 50 μg/mL streptomycin (Sigma, P4333-100 ML).

To generate stable cell lines, HEK293T Flp-In T-REx cells were seeded in six-well plates and grown overnight in antibiotic-free medium. The cells were then cotransfected with a 1:10 ratio of Flp-In expression vector (pRB7, containing 2xFKBP-APEX2-ULK1, or pRB30, containing 2xFKBP-APEX2) to Flp-recombinase plasmid (Thermo Fisher Scientific, V600520). After 24 h, the medium was exchanged with one supplemented with 15 μg/mL Blasticidin (InvivoGen, ant-bl-05). After 24 h, the cells were expanded from six-well plates to p100 dishes (Sarstedt, #83.3902.300) and cultured in media containing 15 μg/mL Blasticidin (InvivoGen, ant-bl-05) and 100 μg/mL Hygromycin B Gold (InvivoGen, ant-hg-1) to select for stable cell lines. The selection medium was exchanged every 5 days. Colonies were then picked and transferred to 24-well plates and expanded. The cells were then checked for doxycycline-inducible target gene expression using Western blotting. All cells were tested for mycoplasma contamination before experiments.

### Antibodies and Ligands Used for Western Blot Analysis

The following primary antibodies were used in this study: mouse monoclonal anti-GFP (1:100, clone 2B6, Merck, cat. no. MABC1689), mouse monoclonal anti-Pgk1 antibody (1:10 000; 22C5D8, Invitrogen), and Streptavidin-HRP (1:3000; #3999, Cell Signaling Technology).

### Standard Biochemical Assays

Yeast cell extracts were incubated with urea loading buffer (120 mM Tris-HCl pH 6.8, 5% glycerol, 8 M urea, 143 mM β-mercaptoethanol, and 8% SDS), boiled, and analyzed by SDS-PAGE. Protein extracts were transferred to nitrocellulose membranes, and proteins were detected by immunoblotting, using the ECL detection system with HRP coupled secondary antibodies.

### Acetylation of Affinity Purification Ligands Using Sulfo-NHS-Acetate

One hundred microliters of magnetic GFP-Trap-MA beads (ChromoTek, catalog number: gtma) and streptavidin magnetic beads (Pierce, Thermo Scientific, catalog number: 88816) were washed three times in 100 μL of reaction buffer (50 mM HEPES-KOH, pH 7.8) and resuspended in 90 μL of reaction buffer. Ten microliters of Sulfo-NHS-Acetate (Pierce, Thermo Scientific, catalog number: 26777) in appropriate dilution was added to achieve a final concentration as indicated and incubated for 60 min at room temperature. The S-NHS-Ac modified beads were washed three times with 50 mM ABC buffer (ammonium bicarbonate, Sigma, 09830-1KG) and resuspended in 100 μL of PBS buffer (pH 6.8, 0.2% Tween 20, 0.02% sodium azide).

### Yeast Cell Extract Preparation and Immunoprecipitation

Cells were harvested by filtration using an AmershamTM ProtranTM 0.45 μm nitrocellulose blotting membrane (GE Healthcare) and frozen in liquid nitrogen. Frozen cells were pulverized in a cryogenic grinder (SPEX Freezer Mill 6770 or 6870, SPEXSamplePrep, Metuchen, NJ), using five rounds of 3 min breakage at 15 Hz and 2 min of cooling, and the powder was stored at −80 °C. Freezer milled yeast powder was resuspended in RLB+ buffer (1x PBS pH 7.4, 10% glycerol, 0.5% Tween-20, 1 mM sodium fluoride, 20 mM β-glycerol, 1 mM PMSF, 1 mM sodium vanadate and protease inhibitor cocktail [Roche]), cell extracts were cleared by centrifugation twice at 5000*g* for 10 min at 4 °C, and the supernatant was transferred to a new microfuge tube each time. For GFP immunoprecipitation, the protein concentration of yeast cell extracts was adjusted to 20 μg/μL in RLB+ buffer. Mock treated or S-NHS-Ac treated GFP-Trap-MA beads (ChromoTek, catalog number: gtma) were incubated with cell extract for 1 h rotating at 4 °C and washed three times with RLB+ buffer. For Western blot analysis, beads were resuspended in a urea loading buffer and boiled for 5 min at 95 °C. For LC–MS analysis, beads were further processed according to the on-bead digestion protocol.

### Mammalian Cell Extract Preparation and Affinity Purification

HEK293T Flp-In T-REx cells were thawed and seeded, and after 24 h the cells were treated with 1 ug/mL doxycycline (Sigma, D9891-10g) to induce the expression of the 2xFKBP-APEX2 or 2xFKBP-APEX2-ULK1 construct. Forty-eight hours postseeding, the cells were treated with 500 uM biotin-phenol (IrisBiotech, 41994-02-9/LS-3500.5000) and incubated for 45 min at 37 °C. To induce the peroxidase activity of APEX2, the cells were treated with 1 mM H_2_O_2_ (Roth, CP26.5) for 1 min.

After the treatment, cells were washed with DPBS (Sigma, D8537-500 ML) and then with quenching buffer (DPBS, 10 mM sodium ascorbate [Sigma, 11140-50G] and 5 mM Trolox [Sigma, 238813-25G]). Cells were scraped, harvested, and lysed by resuspension in RIPA buffer (50 mM Tris, 150 mM NaCl, 0.1% SDS, 0.5% sodium deoxycholate, 1% TritonX-100, 1x protease inhibitor cocktail [Roche, 05056489001]), supplemented with quenching reagents (10 mM sodium ascorbate, 1 mM Trolox). The lysate was cleared by centrifugation at 10 000*g* at 4 °C. The supernatant was incubated for 1 h at 4 °C with mock treated or S-NHS-Ac treated magnetic streptavidin beads (Pierce, 88816). After incubation, beads were washed three times with a RIPA buffer, supplemented with quenching reagents. Beads were resuspended in 1x Laemmli SDS sample buffer (Alfa Aesar, J61337) and boiled for 5 min at 95 °C.

### On-Bead Digestion and Sample Preparation for LC–MS Analysis

**Figure 1:**
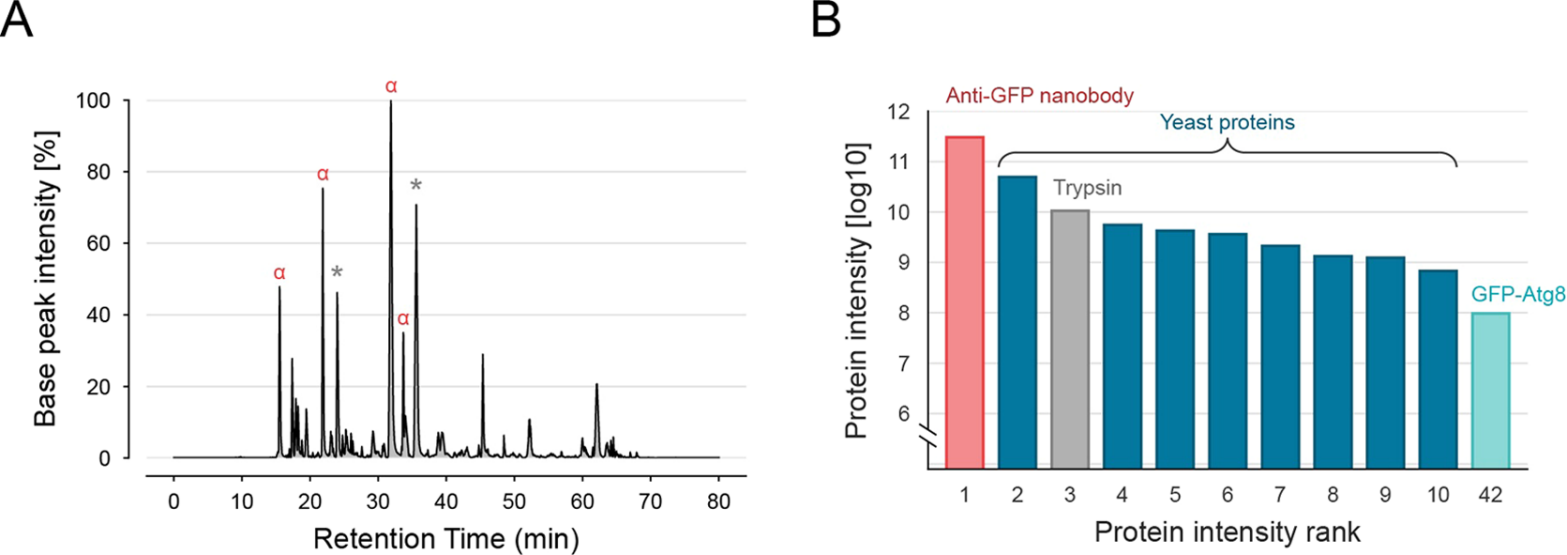
Affinity ligand derived peptides constitute the most abundant signals in the LC–MS analysis after standard tryptic on-bead digestion. GFP-Atg8 was purified from yeast with anti-GFP nanobodies, applying a standard on-bead digestion protocol using trypsin. (A) MS1 base peak chromatogram of the LC–MS analysis. Red α indicates nanobody derived ions, while gray asterisks (*) indicate unassigned ions. (B) Log_10_ protein intensities of the 10 most abundant proteins and of the bait protein GFP-Atg8.

**Figure 2:**
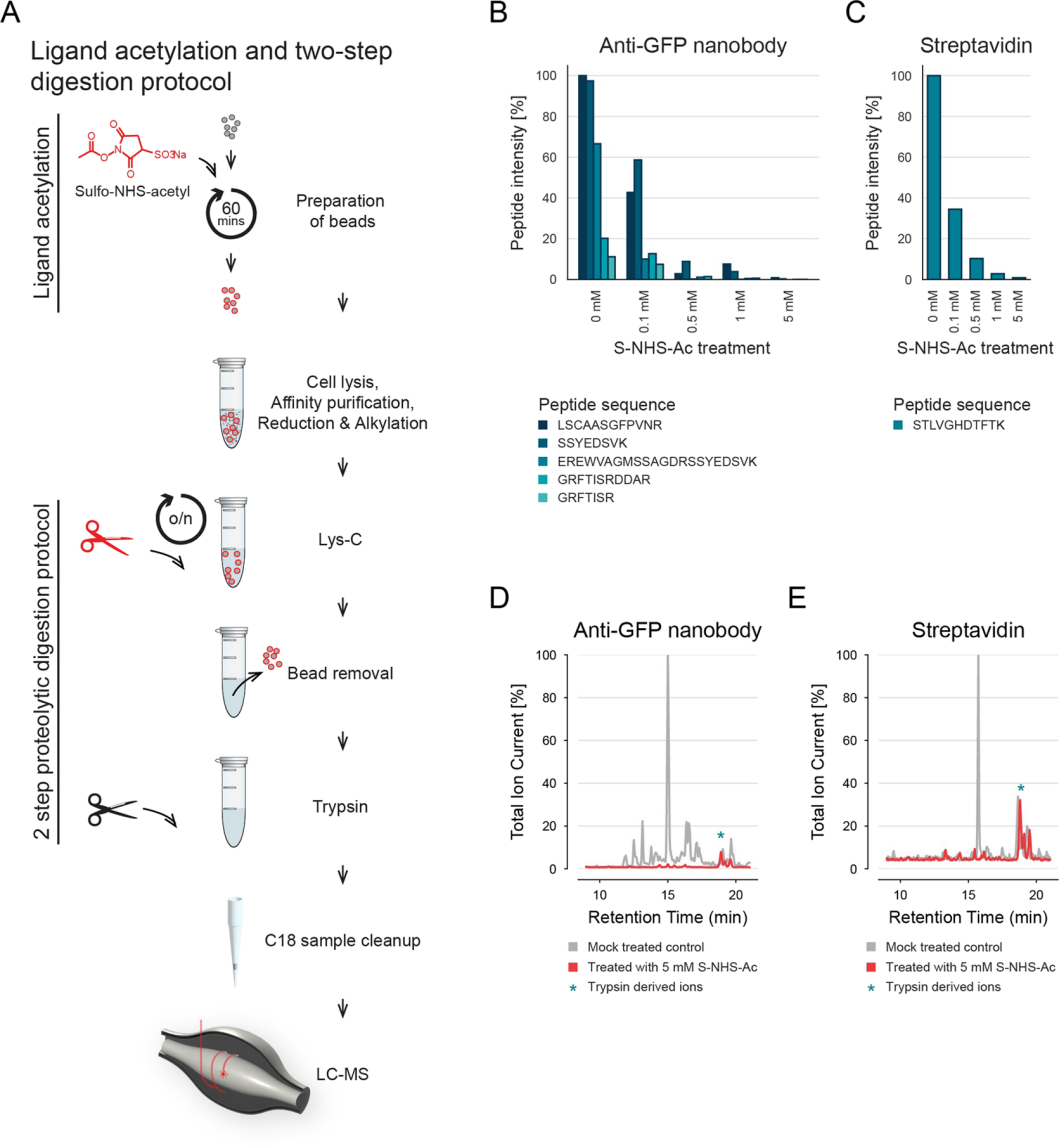
S-NHS-Ac treatment of bead-bound ligands drastically reduces ligand codigestion. (A) Scheme of the presented protocol: Beads are pretreated with S-NHS-Ac before incubation with cell lysate for affinity purification. Next, affinity purified proteins are reduced, alkylated, and eluted by Lys-C treatment. Beads are removed, and the eluate is further digested with trypsin. After sample cleanup using C18 stage tips, peptides are analyzed by LC–MS. (B–E) Anti-GFP nanobody or streptavidin-coated magnetic beads were treated with different concentrations of S-NHS-Ac, subjected to the two-step digestion protocol and analyzed by LC–MS. (B) Relative intensity of the five most abundant anti-GFP nanobody peptides; the average intensity of two replicates is shown. (C) Relative intensity of the most prominent streptavidin peptide; the average intensity of two replicates is shown. (D and E) MS1 TIC chromatogram of LC–MS runs from mock treated beads (gray) or beads treated with 5 mM S-NHS-Ac (red). Turquoise asterisks (*) indicate trypsin derived ions.

After affinity purification, the beads were transferred into fresh 0.2 mL vials (Corning), washed five times with 100 μL of 50 mM ABC buffer (ammonium bicarbonate, Sigma, 09830-1KG), and resuspended in 30 μL of 2 M urea (Sigma) in 50 mM ABC buffer. Beads were reduced and alkylated using either TCEP (tris(2-carboxyethyl)phosphine hydrochloride, Sigma) and MMTS (S-methylmethanethiosulfonate, Fluka) for the S-NHS-Ac titration experiment [Figure 2], or DTT (dithiothreitol, Roche, 10708984001) and IAA (iodoacetamide, Sigma, I6125-5G) for all other experiments. TCEP and DTT were used in final concentrations of 0.2 and 10 mM, respectively, for 30 min at room temperature. MMTS and IAA were used in final concentrations of 1 and 20 mM, respectively, in the dark at room temperature for 15 min. The alkylation reaction was quenched by adding 0.1 mM TCEP or 5 mM DTT. For the classical on-bead digestion protocol [Figure 1], the alkylated proteins were eluted by digestion with 150 ng of Trypsin (Trypsin Gold, Mass Spec grade, Promega, V5280) for 90 min at 37 °C. The supernatant was transferred to a new 0.2 mL vial and further digested overnight at 37 °C by the addition of 150 ng of Trypsin. For the newly developed two-step digestion protocol, the alkylated proteins were eluted by digestion with 150 ng of Lys-C (FUJIFILM Wako Pure Chemical Corp., 125-02543) overnight at 25 °C in the dark. The supernatant was transferred to a new 0.2 mL vial and further digested for 5 h at 37 °C by the addition of 150 ng of Trypsin. For either protocol, the proteolytic digestion was stopped by adding a 10% TFA solution (trifluoroacetic acid, Thermo Scientific, 28903) to a final concentration of 0.5%. Peptide samples were desalted using C18 (Empore, 2215-C18) StageTips as described by Rappsilber et al.,^13^ and peptides were resuspended in 20 μL of 0.1% TFA.

### Mass Spectrometry Analysis

LC–MS/MS analysis was performed on an UltiMate 3000 RSLC nano-HPLC System (Thermo Scientific), containing both a trapping column for peptide concentration (PepMap C18, 5 × 0.3 mm, 5 μm particle size) and an analytical column (PepMap C18, 500 × 0.075 mm, 2 μm particle size (Thermo Scientific)), coupled to a Q Exactive HF-X Orbitrap (with HCD, higher-energy collisional dissociation mode) mass spectrometer via a Proxeon nanospray flex ion source (all Thermo Scientific). For peptide chromatography, the concentration of organic solvent (acetonitrile) was increased linearly over 2 h (for experiments shown in Figures 1 and 4) or over 90 min (for experiments shown in Figure 2) from 1.6% to 28% in 0.1% formic acid or at a flow rate of 230 nL/min. For acquisition of MS2 spectra, the instrument was operated in a data-dependent mode with dynamic exclusion enabled. The scan sequence began with an Orbitrap MS1 spectrum with the following parameters. Experiments shown in Figures 1 and 4: resolution 120 000, scan range 375–1500 *m*/*z*. Experiments shown in Figure 2: resolution 60 000, scan range 380–1650 *m*/*z*. Automatic gain control (AGC) target 3 × 10^6^ and maximum injection time (IT) 60 ms. The top 8 [Figures 1 and 4] or top 10 [Figure 2] precursors were selected for MS2 analysis (HCD) with the following parameters: resolution 30 000, AGC 1 × 10^5^, maximum IT 250 ms [Figure 2] or 200 ms [Figures 1 and 4], isolation window 1.6 *m*/*z* [Figures 1 and 4] or 1.2 *m*/*z* [Figure 2], scan range 200–2000 *m*/*z*, and normalized collision energy (NCE) 27 [Figures 1 and 4] or 28 [Figure 2]. The minimum AGC target was set at 1 × 10^5^, which corresponds to a 4.0 × 10^4^ intensity threshold [Figures 1 and 4], or at 1 × 10^4^, which corresponds to a 5.0 × 10^5^ intensity threshold [Figure 2]. Peptide match was set to preferred. In addition, unassigned, singly, and >6+ charged species and isotopes were excluded from MS2 analysis, and dynamic exclusion was set to 20 s [Figures 1 and 4] or 10 s [Figure 2].

### LC–MS Data Analysis

The RAW MS data were analyzed with FragPipe (19.1), using MSFragger (3.7),^14^ IonQuant (1.8.10),^15^ and Philosopher (4.8.0).^16^ The default FragPipe workflow for label free quantification with match between runs (LFQ-MBR), except that “Normalize intensity across runs”, was turned off. Cleavage specificity was set to Trypsin/P, with two missed cleavages allowed. The protein FDR was set to 1%. A mass of 57.02146 (carbamidomethyl) was used as fixed Cysteine modification, methionine oxidation, and protein N-terminal acetylation were specified as variable modifications. MS2 spectra were searched against the *S. cerevisiae* reference proteome from Uniprot (Proteome ID: UP000002311, release 2022.01) containing 6059 entries, concatenated with a database of 379 common laboratory contaminants (in house database) and one entry for the anti-GFP nanobody sequence extracted from the RCSB Protein Data Bank (PDB ID: 3OGO(17)). For the S-NHS-Ac titration experiment [Figure 2], only the contaminants database, also containing the streptavidin and the anti-GFP nanobody sequences, was used; the protein FDR was set to 5%; and a mass of 45.98772 (methylthio) was used as fixed Cysteine modification.

Computational analysis was performed using Python and the in-house developed python libraries MsReport (version 0.0.14), XlsxReport (version 0.0.6), and Maspy (version 1.1.3). The experiment shown in Figure 1 included only one LC–MS run; hence raw protein intensities reported by FragPipe were used for the analysis. Raw protein intensities correspond to the sum of all peptide ion intensities mapped to a specific protein after razor assignment. For the S-NHS-Ac titration experiment shown in Figure 2, not normalized LFQ peptide intensities from FragPipe were used for the analysis. The experiments shown in Figure 4, using mock treated or S-NHS-Ac treated anti-GFP nanobody-coated beads, were analyzed and processed separately as follows. Only noncontaminant proteins, proteins identified with at least two peptides, and proteins that were quantified in at least two replicates of one experiment were considered for the analysis. LFQ protein intensities reported by FragPipe were log2-transformed and normalized across samples using the ModeNormalizer from MsReport. This method involves calculating log2 protein ratios for all pairs of samples and determining normalization factors based on the modes of all ratio distributions. Missing values were imputed by drawing random values from a normal distribution. Sigma and mu of this distribution were calculated per sample from the standard deviation and median of the observed log2 protein intensities (μ = median sample LFQ intensity – 1.8 standard deviations of the sample LFQ intensities, σ = 0.3 × standard deviation of the sample LFQ intensities). For generating TIC and base peak chromatograms, RAW files were converted to mzML using msConvert from the open source software Proteowizard (version 3.0.22155).^18^ Peak picking was enabled on the MS1 and MS2 levels with the “Prefer Vendor” option. TIC and base peak intensities were extracted from the mzML files with the Maspy library and visualized using Python.

## Results

### Conventional On-Bead Digestion Leads to Ligand Peptide Contamination

To demonstrate the problem of ligand codigestion, we performed an exemplary AP–MS experiment with anti-GFP nanobody-coated magnetic beads and a *Saccharomyces cerevisiae* strain expressing a GFP-tagged version of the autophagy related protein Atg8. After affinity purification of GFP-Atg8, bead-bound proteins were eluted by 90 min on-bead digestion with trypsin, followed by removal of the beads and subsequent overnight digestion of the supernatant with trypsin. LC–MS analysis revealed that ligand-derived peptides constituted the most abundant signals in the MS1 base peak chromatogram [Figure 1A]. Moreover, the protein intensity of the anti-GFP nanobody was more than one order of magnitude higher as compared to yeast proteins [Figure 1B and Table S1]. Consequently, the high amount of codigested ligand strongly limited the amount of sample that could be subjected to LC–MS analysis.

### S-NHS-Ac Bead Modification and Two-Step Digestion Reduces Ligand Peptide Contamination

Acetylation of lysine has been reported to block cleavage by trypsin and Lys-C proteases, most likely due to steric hindrance and loss of the positive charge on the ε-amine group. Pretreatment of beads with an agent capable of efficiently acetylating lysine residues should therefore confer Lys-C protease resistance to bead-bound ligands. Using chemically acetylated beads would allow the elution of enriched proteins by on-bead digestion with Lys-C while preventing leaching of ligand through codigestion. In a second step, the supernatant could then be separated from the beads and be digested with trypsin to generate standard tryptic peptides. For this purpose, an ideal acetylating agent should be active in mild buffer conditions that do not compromise ligand integrity, and the acetylation reaction should be fast and simple to carry out. Furthermore, the agent should be of low cost, not toxic, and be readily available to any research laboratory. We identified Sulfo-NHS-Acetate (S-NHS-Ac) as being an optimal candidate for our purpose that fulfills all of these requirements [Figure 2A].

To test the outlined ligand-acetylation two-step digestion protocol, we incubated anti-GFP nanobody-coated and streptavidin-coated beads for 60 min with different concentrations (0.1–5 mM) of S-NHS-Ac and compared the amount of ligand derived peptides to mock treated beads using label-free LC–MS. The intensity of ligand derived peptides was reduced to below 50% at the lowest concentration of 0.1 mM S-NHS-Ac. Increasing the reagent concentration led to a successive decrease of detected ligand signals, demonstrating that lysine acetylation by S-NHS-Ac effectively prevents Lys-C mediated peptide cleavage [Figure 2B and C and Table S2]. Treatment with 5 mM S-NHS-Ac resulted in nearly complete proteolytic protection of the ligands, as shown by the reduction of the respective signals in the MS1 total ion current (TIC) chromatogram [Figure 2D and E]. Therefore, we selected this concentration for the subsequent ligand modification procedure.

### Acetylation of Ligand Does Not Affect Substrate Binding Capacity

**Figure 3:**
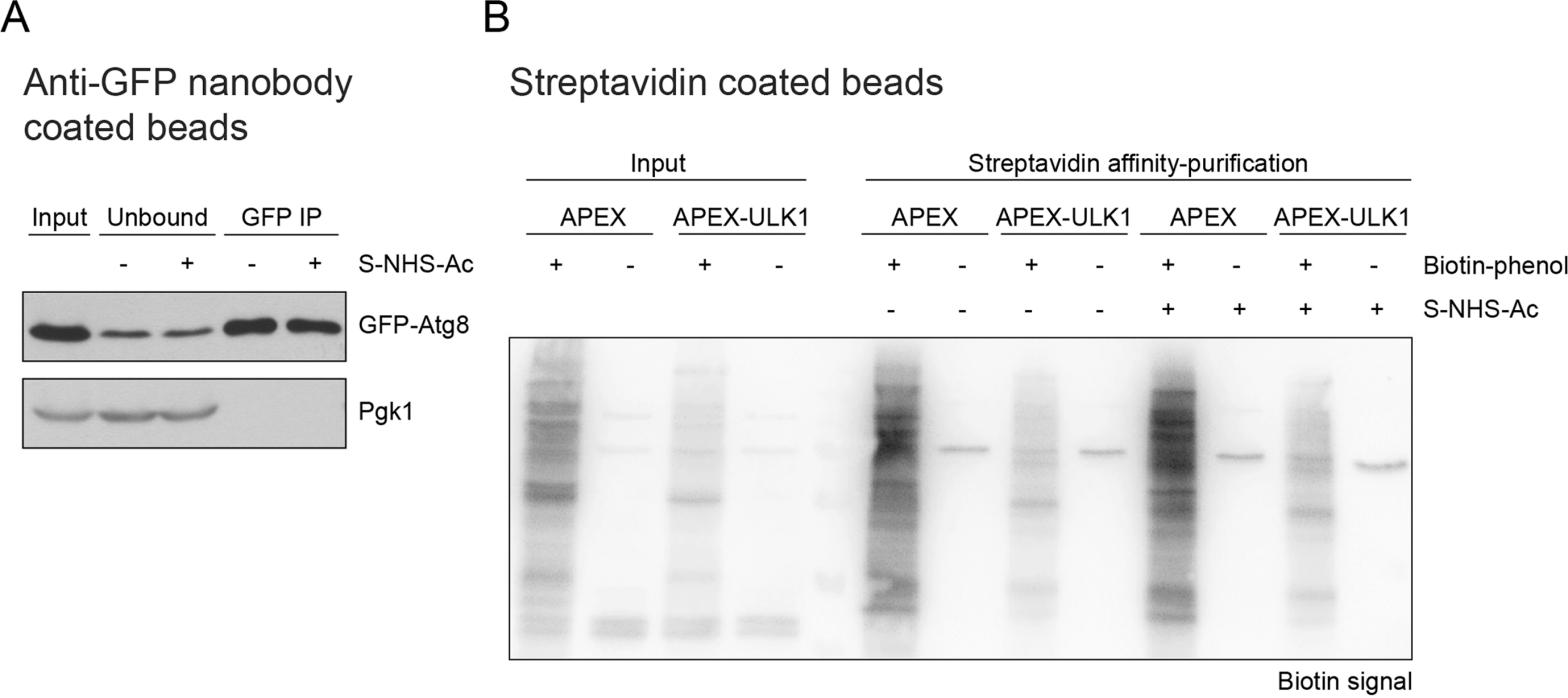
S-NHS-Ac treatment of bead-bound ligands does not affect substrate binding capacity. (A) Western blot analysis of GFP-Atg8 immunoprecipitations using mock treated anti-GFP nanobody beads (−) or beads treated with 5 mM S-NHS-Ac (+). Input: Whole cell extract prepared from *S. cerevisiae* cells expressing GFP-Atg8. Unbound: Signal corresponding to the remaining GFP-Atg8 in the supernatant. Pgk1: Loading control for whole cell extract. (B) Western blot analysis of affinity purifications of biotinylated proteins from human HEK cells expressing the autophagy related protein ULK1 fused to FKBP-APEX (APEX-ULK1) or only FKBP-APEX. All cells were treated with H_2_O_2_, and either with or without biotin-phenol to induce biotinylation of proteins in proximity to APEX. Affinity purification was performed using mock treated streptavidin beads (−) or beads treated with 5 mM S-NHS-Ac (+).

To assess the impact of S-NHS-Ac treatment on the ligand binding capacity, we conducted AP experiments and compared protein yields from mock-treated and S-NHS-Ac treated beads via Western blotting. Specifically, we used magnetic anti-GFP nanobody beads to purify GFP-tagged Atg8 from *S. cerevisiae* cells and found similar amounts of bait protein purified with modified and unmodified beads [Figures 3A and S1A,B]. Similarly, we investigated the effect of S-NHS-Ac treatment on streptavidin beads using human HEK cells expressing FKBP-APEX (FK506-binding protein fused to the engineered ascorbate peroxidase APEX) or FKBP-APEX-ULK1 (FKBP-APEX fused to the autophagy related protein ULK1). In the presence of hydrogen peroxide (H_2_O_2_) and biotin-phenol, APEX is capable of proximity-dependent biotinylation of proteins by generating biotin-phenol radicals that facilitate the covalent labeling of neighboring amino acid residues with biotin. We used acetylated or unmodified magnetic streptavidin beads to purify biotinylated proteins, from cells treated with or without biotin-phenol. Using Western blotting to detect biotinylated proteins, we observed no difference in protein yield between the mock-treated and S-NHS-Ac-treated streptavidin beads [Figures 3B and S2]. Taken together, these results demonstrate that the chemical acetylation does not affect the binding capacity of anti-GFP nanobodies and streptavidin.

### S-NHS-Ac Bead Modification and Two-Step Digestion Enhances Sensitivity for AP–MS

**Figure 4:**
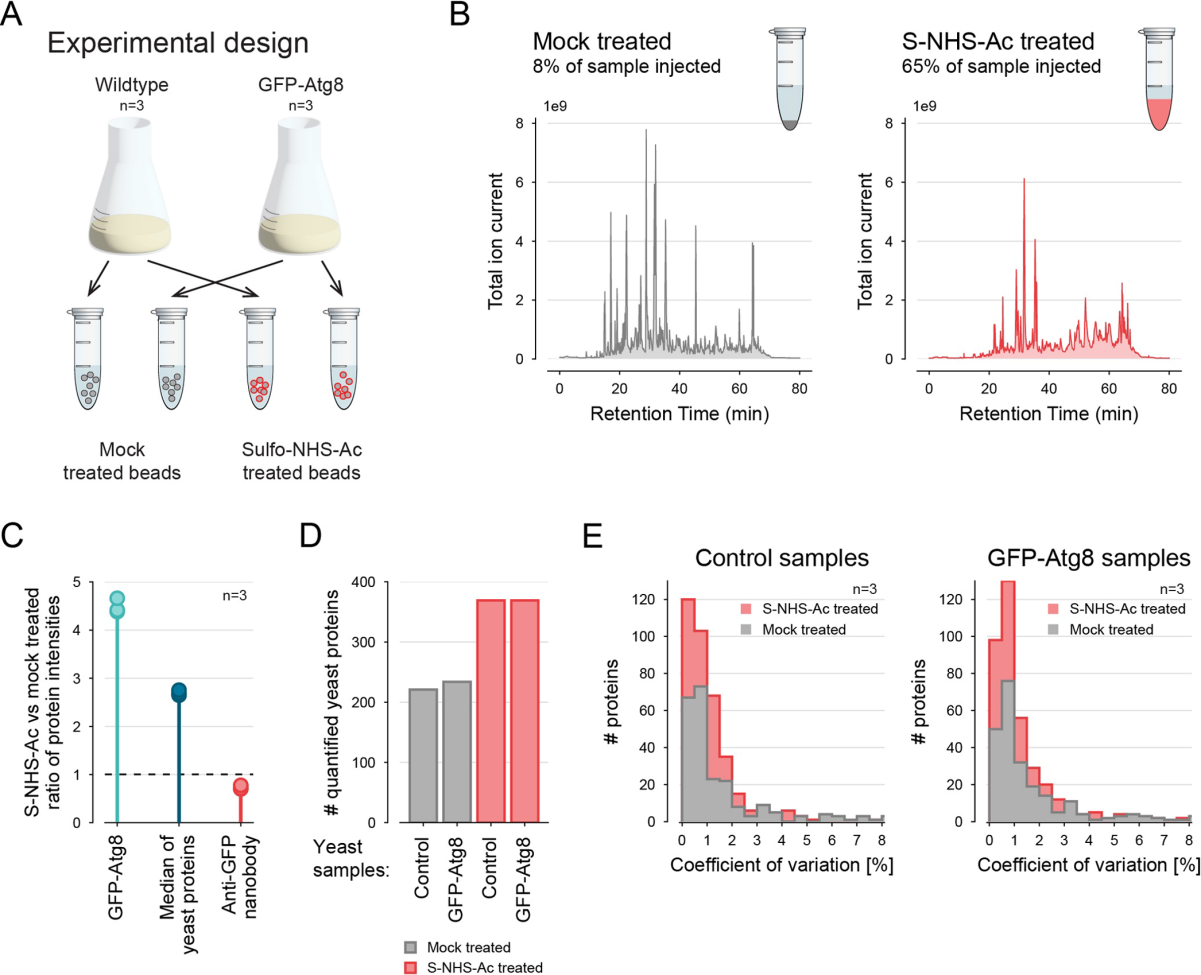
Enhanced sensitivity for AP–MS using S-NHS-Ac bead modification and two-step digestion. (A) Scheme of the experimental setup. GFP-tagged Atg8 was purified from *S. cerevisiae* using anti-GFP nanobody-coated beads that were either not treated (mock) or treated with 5 mM S-NHS-Ac. Experiments were performed in three replicates. (B) MS1 TIC chromatogram of GFP-Atg8 immunoprecipitations performed with untreated and S-NHS-Ac treated anti-GFP nanobody-coated beads. Note that different sample amounts were injected for MS analysis. The second replicates of the GFP-Atg8 samples are shown. (C) Protein intensities from the GFP-Atg8 samples were compared between the immunoprecipitations from mock treated and S-NHS-Ac treated beads. Ratios were calculated between replicate pairs (replicate 1 vs 1, 2 vs 2, 3 vs 3). For calculating the median ratio of yeast proteins, only proteins quantified in all three replicates were considered. (D) The number of yeast proteins quantified in at least two replicates per condition is shown. (E) Comparison of the coefficient of variation of protein intensities between replicates from immunoprecipitations of wild type (left) and GFP-Atg8 containing samples (right).

We next wanted to test our S-NHS-Ac-based AP–MS protocol under realistic experimental conditions. Specifically, we purified GFP-tagged Atg8 from *S. cerevisiae* cells in three replicates and compared it to a purification with untagged Atg8 [Figure 4A]. To test the performance of our ligand-acetylation protocol, we performed the experiment with S-NHS-Ac treated and mock treated beads and applied the two-step digestion protocol for LC–MS sample preparation [Figure 2A].

To obtain high sensitivity in LC–MS analysis, it is important to load as much of the sample as feasible. Nonetheless, the intensity of the most abundant peptide ion imposes a restriction on the amount that can be introduced into the system without overloading it. To assess optimal sample amounts that could be subjected to the LC–MS system, we quantified peptide amounts using UV-chromatograms on a monolithic LC–UV system. As expected, we found that pull downs with S-NHS-Ac treated beads exhibited strongly reduced UV signals (∼1/8), indicating a significant reduction in the ligand-derived background. Accordingly, we injected 65% of the samples purified with S-NHS-Ac treated beads and only 8% of the samples derived from the control experiment (untreated beads) to LC–MS analysis. Indeed, we observed similar maximum MS1 TIC intensities in both experiments [Figure 4B]. Moreover, we observed up to 4-fold higher protein intensities for GFP-Atg8 and other yeast proteins and a significantly better anti-GFP nanobody to bait ratio in the sample purified with S-NHS-Ac-treated beads [Figure 4C]. Note that the disparity of the expected 8-fold change and the observed 4–5-fold change of Atg8 stems from a drop of LC–MS performance during the measurement of the samples purified with S-NHS-Ac treated beads, and not due to a reduction of nanobody binding capacity [Figures 3 and S3]. Nevertheless, the higher injection amounts resulted in quantification of 50% more proteins [Figure 4D and Table S3]. Moreover, we detected more proteins with a low coefficient of variation among replicates of the GFP-Atg8 samples and among replicates of the untagged samples, demonstrating improved quantification in the experiment performed with S-NHS-Ac treated beads [Figure 4E]. Interestingly, the median intensity increase of yeast proteins was less pronounced as compared to GFP-Atg8 [Figure 4C]. This could suggest that S-NHS-Ac treatment affects bead matrix properties, potentially reducing nonspecific protein binding. However, reliable conclusions would necessitate further testing across various bead matrixes and lysates from diverse organisms.

To conclude, we successfully developed a simple and fast protocol that prevents contamination of AP–MS samples with high amounts of ligand-derived peptides. Our findings reveal that the S-NHS-Ac treatment of beads does not compromise the binding efficiency of anti-GFP nanobody and streptavidin beads, while simultaneously rendering them resistant to proteolysis by Lys-C. Furthermore, we have demonstrated that by minimizing ligand contamination through the application of our protocol, we can achieve higher sample loading amounts, resulting in enhanced sensitivity and improved quantitative accuracy in LC–MS analysis.

## Discussion

The present study presents a novel protocol for preventing the problem of ligand contamination in AP–MS experiments, based on chemical acetylation of the bead-bound ligand and elution of bait proteins by stepwise proteolytic digestion. What sets this protocol apart from previous methods is its simplicity and ease of use, which make it highly accessible and user-friendly. The preparation of the beads takes approximately 1 h, uses only standard buffers and one nontoxic reagent, and is essentially free of harsh chemical conditions, supporting ligand integrity. Therefore, our two-step digestion protocol is well compatible with typical AP–MS workflows, performing the affinity purification during the day, Lys-C digestion overnight, followed by a 5 h trypsin digestion and a desalting step on the next day prior to LC–MS analysis. We have made a step-by-step user guide available on protocols.io to allow the easy application of our protocol.^19^

We demonstrate that the application of this protocol leads to a strong reduction of ligand-derived peptide contaminations while preserving the bait binding capacity of anti-GFP nanobody and streptavidin beads. In principle, the protocol is applicable to any proteinaceous ligand. However, it is important to exercise caution while applying the developed protocol to a novel ligand, as modification of lysines that are important for substrate interaction can impair its binding capability. Another important point to consider is that our protocol does not involve covalent linking of ligands to the matrix or between multiple ligand subunits. This can lead to leaching of classical IgG antibodies or other ligands attached to the matrix with disulfide bonds. To prevent this, chemical reduction is performed after the Lys-C digest, at peptide level, once the ligand-containing matrix has been removed from the sample.

Several methods have been employed to elute bait proteins in conjunction with AP–MS, each with its own advantages and disadvantages. One example involves stabilizing the ligands to the matrix through cross-linking in combination with chemical elution of the bait protein. However, not all ligands tolerate the cross-linking procedure, and elution procedures either are not efficient^20^ or interfere with downstream MS analysis.^21^ To overcome the issue of inefficient elution, a two-step digestion procedure has been combined with cross-linked antibodies. After affinity purification, bait proteins are eluted by a short predigestion with Lys-C. Next, the ligand matrix is removed, and tryptic digestion is continued for 16 h.^20^ Despite the efficient bait elution and substantial reduction in ligand elution offered by this protocol, it still utilizes cross-linking, which exposes ligands to harsh chemical environments and extensive chemical modifications. Two recent protocols employ chemical derivatization of the ligand to create trypsin resistance.^10,11^ However, similar to chemical cross-linking, both derivatization procedures expose the ligand to harsh chemical conditions. Furthermore, as both methods modify arginines and lysines, two of the three positively charged amino acids, there is an additional risk of altering the ligand’s binding site, which may compromise its ability to bind the epitope.

By combining chemical derivatization of lysines under mild conditions with a two-step digestion protocol, our approach combines several advantages of the above-mentioned sample processing strategies. It allows efficient bait elution, protects from contamination with ligand–peptides, uses nontoxic, mild chemical conditions, and is simple to carry out. We have adopted the protocol as a standard procedure in our core facility, and it has been successfully used in several projects.^22,23^ In conclusion, the protocol presented in this study offers a practical and efficient solution for preventing ligand contamination in AP–MS samples with potential applications in a variety of research settings.

## Data Availability

The mass spectrometry proteomics data have been deposited to the ProteomeXchange Consortium via the PRIDE^24^ partner repository with the data set identifier PXD043709. The Python scripts used for data processing and analysis are available from our GitHub repository at https://github.com/maxperutzlabs-ms/Publication_Resources in the folder “2023_hollenstein_chemical-ligand-acetylation”.

## Supporting Information Available

Figures S1 and S2 containing Western blot replicates and uncropped images related to Figure 3; Figure S3 containing supplemental information related to Figure 4 (PDF)
Table S1: MS protein quantification related to Figure 1 (XLSX)
Table S2: MS protein and peptide quantification related to Figure 2 (XLSX)
Table S3: MS protein quantification related to Figure 4 (XLSX)
