# Supplementary material for: Chemical Acetylation of Ligands and Two-Step Digestion Protocol for Reducing Codigestion in Affinity Purification–Mass Spectrometry
Source: J Proteome Res. 2023 Sep 15;22(10):3383–91. doi: 10.1021/acs.jproteome.3c00424 (PMC10563155; doi:10.1021/acs.jproteome.3c00424)
Supplement: Supplementary file 1 — pr3c00424_si_001.pdf [file pr3c00424_si_001.pdf]

Chemical acetylation of ligands and two-step digestion protocol for reducing co-digestion in affinity purification-mass spectrometry

David M. Hollenstein[1,2], Margarita Maurer-Granofszky[3], Wolfgang Reiter[2], Dorothea Anrather[2], Thomas Gossenreiter[2], Riccardo Babic[4,5,6], Natascha Hartl[1,2], Claudine Kraft[4,7], Markus Hartl[1,2]*

[1]Department for Biochemistry and Cell Biology, University of Vienna, Center for Molecular Biology, Vienna Biocenter Campus (VBC), Dr. Bohr-Gasse 9, 1030 Vienna, Austria

[2]Mass Spectrometry Facility, Max Perutz Labs, Vienna Biocenter Campus (VBC), Dr. Bohr-Gasse 7, 1030 Vienna, Austria

[3]St. Anna Children's Cancer Research Institute (CCRI), Zimmermannplatz 10, 1090 Vienna, Austria

[4]Institute of Biochemistry and Molecular Biology, ZBMZ, Faculty of Medicine, University of Freiburg, 79104 Freiburg, Germany

[5]Faculty of Biology, University of Freiburg, 79104 Freiburg, Germany

[6]Spemann Graduate School of Biology and Medicine (SGBM), University of Freiburg, 79104 Freiburg, Germany

[7]CIBSS - Centre for Integrative Biological Signalling Studies, University of Freiburg, 79104 Freiburg, Germany

*** Corresponding author information:**

Markus Hartl, Max Perutz Labs, Mass Spectrometry Facility, Dr. Bohr-Gasse 9, 1030 Vienna, Austria; Phone: +43-1-4277-52846; E-Mail: markus.hartl@univie.ac.at

Table of Content

Supplemental table legends .... page 2

Supplemental figure 1 .... page 3

Supplemental figure 2 .... page 4

Supplemental figure 3 .... page 5

Supplemental Table Legends

Supplemental Excel table 1: MS protein quantification related to Figure 1:
Excel tables listing the MS protein quantification results of experiments shown in Figure 1

Supplemental Excel table 2: MS protein and peptide quantification related to Figure 2:
Excel tables listing the MS protein and peptide quantification results of experiments shown in Figure 2

Supplemental Excel table 3: MS protein quantification related to Figure 4:
Excel tables listing the MS protein quantification results of experiments shown in Figure 4

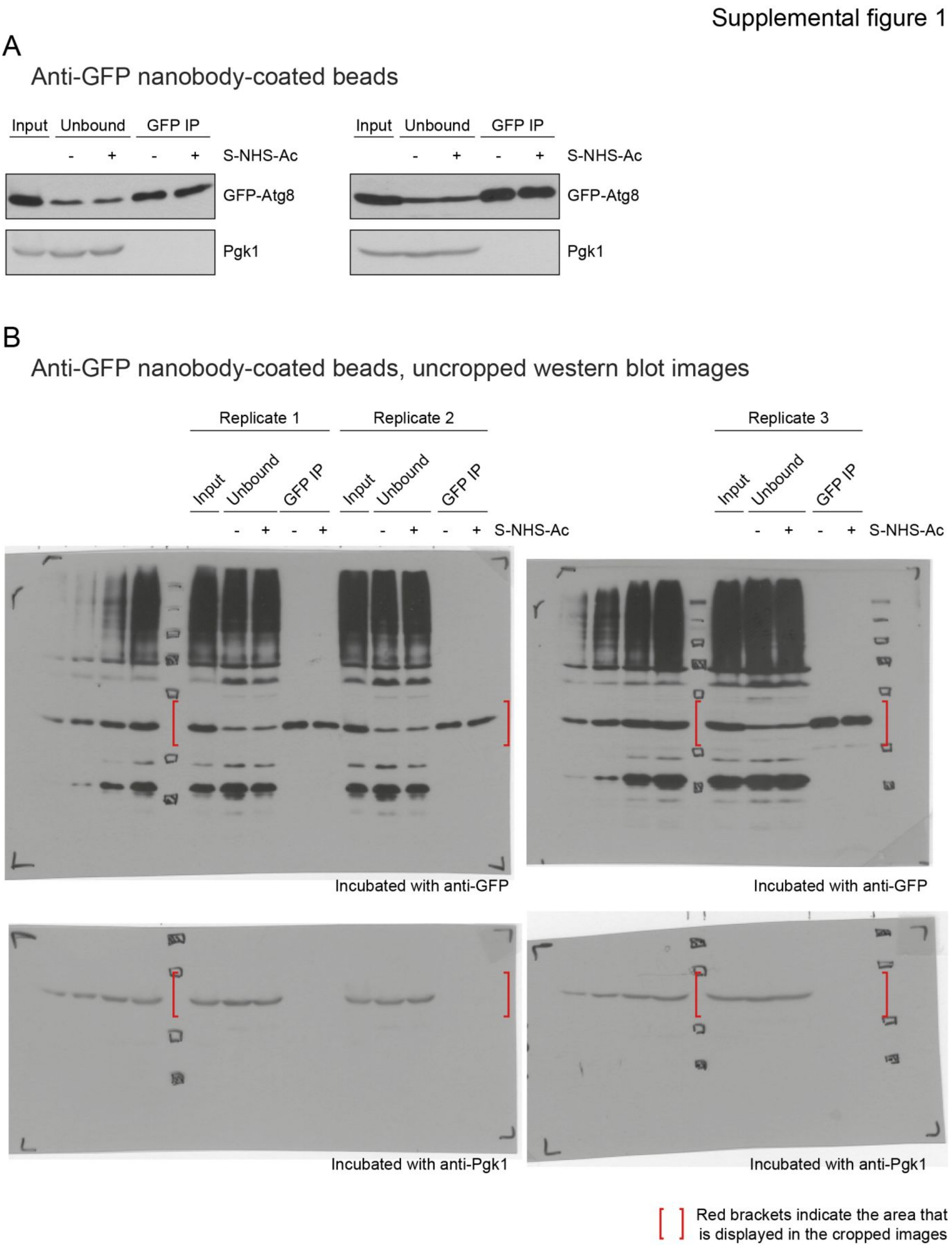

Supplemental figure 1: Supplement to figure 3A showing replicate 2 and 3 and uncropped western blot images.

A and B, Western blot analysis of GFP-Atg8 immunoprecipitations using mock treated anti-GFP nanobody beads (-) or beads treated with 5 mM S-NHS-Ac (+). Input: whole cell extract prepared from *S. cerevisiae* cells expressing GFP-Atg8. Unbound: signal corresponding to the remaining GFP-Atg8 in the supernatant. Pgk1: loading control for whole cell extract. **A,** Replicate 2 and 3 of western blots shown in figure 3A. **B,** Uncropped western blot images.

Supplemental figure 2

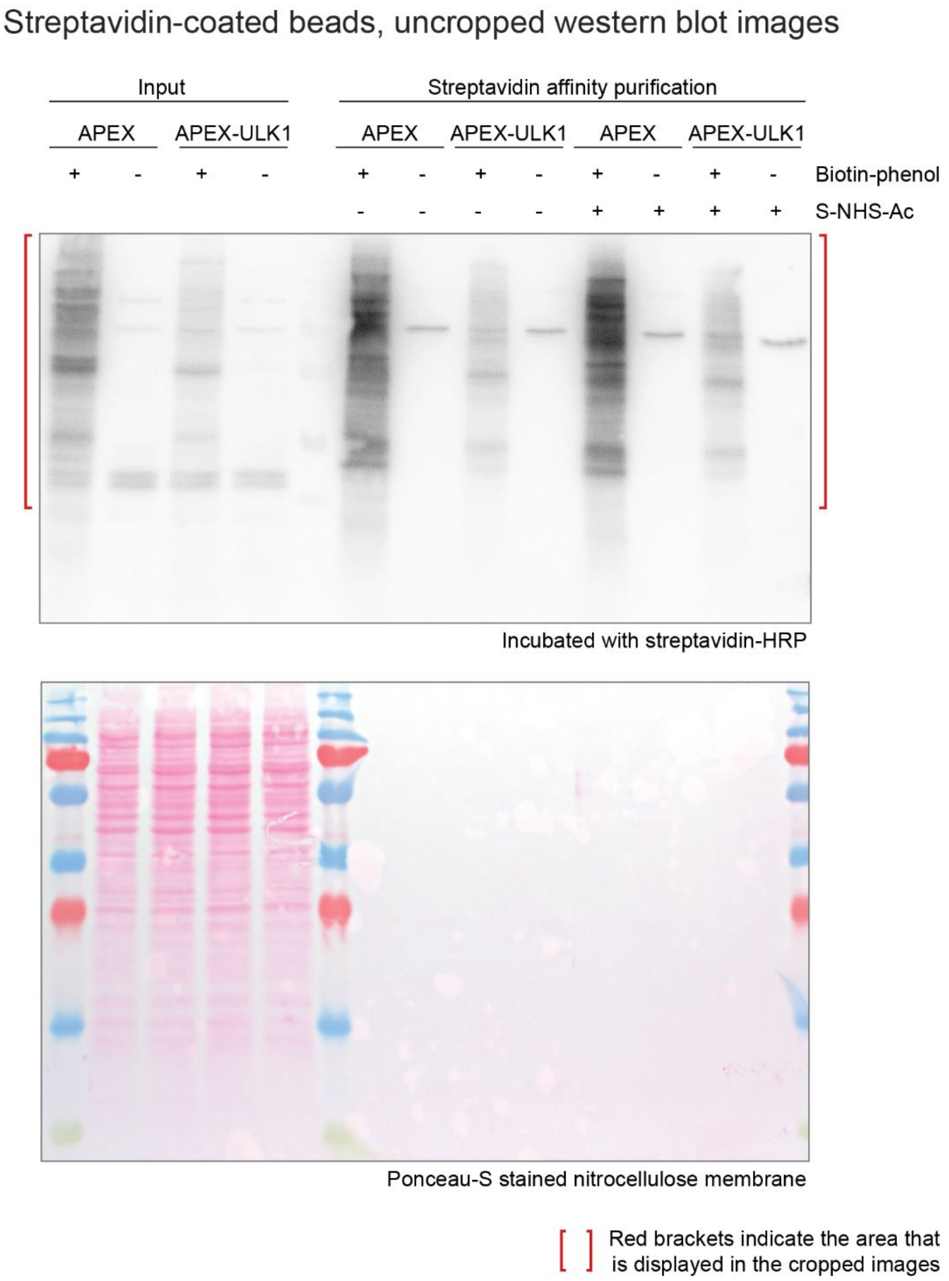

Supplemental figure 2: Supplement to figure 3B showing uncropped western blot images.

Western blot analysis of affinity purifications of biotinylated proteins from human HEK cells expressing the autophagy related protein ULK1 fused to FKBP-APEX (APEX-ULK1) or only FKBP-APEX. All cells were treated with H_2O_2, and either with or without biotin-phenol to induce biotinylation of proteins in proximity to APEX. Affinity purification was performed using mock treated streptavidin beads (-) or beads treated with 5 mM S-NHS-Ac (+).

Supplemental figure 3

Average LFQ intensity

	S-NHS-Ac treated beads	Mock treated beads
GFP-Atg8	2.15E+09	4.79E+08
Trypsin	1.34E+10	2.82E+09

Ratio GFP-Atg8 vs Trypsin

S-NHS-Ac treated beads	Mock treated beads
0.161	0.170

Relative difference of "GFP-Atg8 vs Trypsin ratio" between the mock treated and S-NHS-Ac treated = **94.44%**

Supplemental figure 3: S-NHS-Ac treatment does not affect the binding capacity of the anti-GFP nanobody, supplement to figure 4.

Average LFQ intensities of GFP-Atg8 and trypsin are depicted for samples purified using S-NHS-Ac treated (red) beads and mock treated (gray) beads. Lower panel: shows ratio of GFP-Atg8 to trypsin intensities. **Note:** In the experiments depicted in Figure 4, affinity purification and MS sample preparation for all 12 samples were performed in parallel to minimize technical variability. This approach maintained consistent quantities of yeast cell extract, beads, and chemical reagents, including a uniform amount of trypsin per sample. By comparing the GFP-Atg8 to trypsin ratio between samples purified using S-NHS-Ac treated beads and mock treated beads, we assessed whether the anti-GFP nanobody's binding capacity was influenced by the treatment. This analysis revealed that the average GFP-Atg8 to trypsin ratio differed by less than 6%, confirming that S-NHS-Ac treatment does not compromise nanobody integrity.
